# A High-Performance Rectangular Gate U Channel FETs with Only 2-nm Distance between Source and Drain Contacts

**DOI:** 10.1186/s11671-019-2879-0

**Published:** 2019-02-04

**Authors:** Xi Liu, Zhengliang Xia, Xiaoshi Jin, Jong-Ho Lee

**Affiliations:** 1 School of Information Science and Engineering, Shenyang University of Technology, Shenyang, 110870 China; 2 School of EECS Eng. and ISRC (Inter-University Semiconductor Research Center), Seoul National University, Shinlim-Dong, Kwanak-Gu, Seoul, 151-742 Korea

**Keywords:** Rectangular gate U channel, Extreme integration, Quantum simulation

## Abstract

A novel high-performance rectangular gate U channel FET (RGUC FET) for extreme integrated distance between source and drain contacts is proposed in this paper. The RGUC FET represents nearly ideal subthreshold characteristics till the distance between source/drain (S/D) contacts reduced to 2 nm. Different from the other recessed or U-shaped channel-based FETs, the gate contacts do not need to be formed in the recessed region but only in a layer of spacer for the insulation between the two vertical parts on both sides of the U channel. Its structural advantages make it possible to be applied to manufacture integrated circuits with higher integration for extreme integrated distance between source and drain contacts. The electrical properties of the RGUC FET were scrupulously investigated by studying the influence of design parameters including the horizontal distance between S/D contacts, the extension height of S/D region, and the thickness and material of the gate oxide layer. The electrical properties of the RGUC FET are verified by quantum simulation. Compared to the other non-planner channel multi-gate FETs, the novel RGUC FET is suitable for higher integration.

## Introduction

As one of the most promising device used in nano-scale integrated circuits (IC), the junctionless field-effect transistor (JL FET) which presents remarkable electrical characteristics compared to conventional junction-based metal oxide semiconductor (MOS) FETs, in addition to its simplicity of fabrication, has been deeply studied in recent years [1–4]. While increasing the gate voltage forms the accumulation region in the channel, resulting to greater on current [5], the introduction of the multiple-gate (MG) FET strengthened the controllability of the source-to-drain current from the gate voltage, resulting to much better subthreshold properties of the device. The junctionless multiple-gate (JL MG) FETs also have been widely studied for years [6–8]. Although the vertical channel gate-all-around MOSFET shows a nearly ideal *I*-*V* performance with a radius only several nanometers, the vertical channel of it makes the source and drain contact could not be manufactured in the same layer, which makes the layout of ICs incompatible with the planner technology. Moreover, as the semiconductor fabrication has been forced to scale down the channel length to be less than 10 nm, the MG FETs face the short-channel effect again [9–11]. In order to overcome the short-channel effect, recessed channel MOSFETs become a hot topic in recent years [12–16]. The modeling and simulation work of recessed channel MOSFETs is also comprehensively carried out [17–20]. A recessed channel MOSFET has both planner vertical channel parts under both source and drain contacts and a horizontal planar channel part. It actually prolonged the effective channel length compared to conventional MOSFETs with only the horizontal planar channel. For the device with the same distance between source and drain contacts, it can be more immune to the short-channel effect compared to conventional MOSFETs with planar channel; however, the experimental data shows that the subthreshold swing of MOSFETs with recess channel can not realize an ideal subthreshold swing with sub 100-nm effective channel length. That is because although the channel length is prolonged, the gate controllability is not strengthened as MG FETs. It should be noted that, it is better to define a new key geometrical parameter related to the description of integration, instead of the channel length. The distance between source and drain contacts is more realistic and effective because the final goal of the design of the nano-scale device is the realization of the best performance in a limited given chip area, and the actual device size is related to the channel width and the distance between source and drain contacts. In order to combine the advantages of both the MG FETs and recessed channel MOSFETs, in our previous work, we proposed saddle-shaped gate FETs with a U-shaped channel [21–23], which promotes the gate controllability to the horizontal channel part of the recessed channel from a planar single-gate type to a 3-D triple-gate type. After that, we upgrade this 3-D triple-gate feature formed not only in the horizontal channel part but also in both vertical channel parts. This device is named as H gate U channel FETs, and the recessed channel is correspondingly upgraded to a 3-D U-shaped tube channel too [24]. As mentioned above, the final goal of the design of the nano-scale device is the realization of the best performance in a limited given chip area through optimization. To realize an optimized high-performance device, both gate structure and the corresponding channel structure should be well considered and designed. Also the fabrication complexity should be considered well. The devices mentioned above such as the recessed channel device, the previously proposed saddle FETs, and HGUC FETs have a common ground, a sandwich structure of gate oxide/gate/gate oxide should be well formed in the small recessed region. This structural feature limits its further promotion of integration. It seems that a good way to promote the integration is to simplify the structural feature in the recessed region and maintain the gate control ability to the vertical channel part and horizontal channel part of the U-shaped channel at the same time. In order to realize these device features and functions, in this paper, we proposed a novel rectangular gate U channel FET (RGUC FET) for extreme integrated distance between source and drain contacts. It has a U-shaped channel which can prolong the effect channel length without increasing the distance between source and drain contacts. Compared to the other U-shaped channel FETs, the RGUC FET is with a simpler inner structure in the recessed region of the U-shaped channel; thereafter, it can realize simpler manufacture in the inner part of the recessed region and smaller distance between source and drain contacts (higher integration). The proposed structure has better gate controllability and smaller reverse leakage current accompanied with higher *I*_ON_/*I*_OFF_ ratio. The distance between source contact and drain contact can be scaled down to less than 2 nm. The whole electric properties are analyzed by quantum simulations.

## Methods

**Fig. 1:**
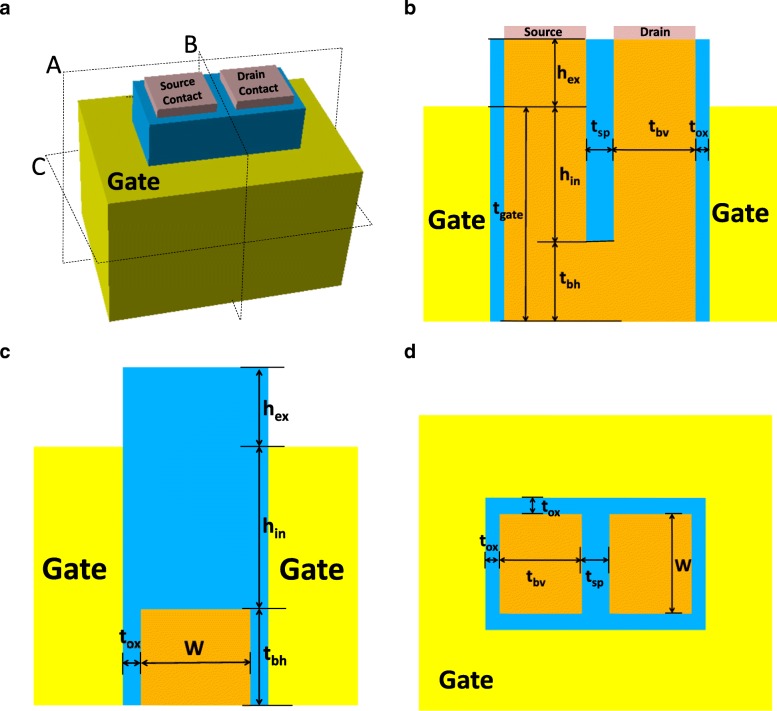
**a** 3D schematic view of the RGUC FET. **b** Profiles of the device cut through plane A of **a**. **c** Profiles of the device cut through plane B of **a**. **d** Profiles of the device cut through plane C of **a**

Figure 1a presents the 3D schematic view of the RGUC FET, and Fig. 1b to d are profiles of the device cut through planes A, B, C, and D shown in Fig. 1a. *W* is the body width of the silicon, *t*_b_ is the body thickness of the silicon, *h*_in_ is the inner height of the spacer in the recessed region, *h*_ex_ is the height of the extension source/drain region, *t*_ox_ is the thickness of the gate oxide around the silicon body, and *t*_sp_ is the spacer thickness of the insulator layer deposited in the recessed region of the U-shaped channel which equals to the distance between source contact and drain contact.

Since the silicon body thickness is less than 6 nm, quantum simulations are introduced in this paper instead of classical simulations to obtain more precise simulation results. All simulations are performed using the TCAD of SILVACO Atlas 3D device simulation, using the concentration-dependent mobility model, concentration-dependent Shockley-Read-Hall model, Auger recombination model, bandgap narrowing model, standard band-to-band tunneling model, and Bohm quantum potential model [25]. The simulation parameters are listed in Table 1. The two vertical body parts are actually cubes with four sides, the top surfaces of which are covered with the source or drain region and the bottom surface are both connected to the horizontal body part. The outer triple sides of the vertical body parts are surrounded by the gate oxide and rectangular gate contact, and the other inner side is connected to the inner spacer in the recessed region. The four sides of the horizontal body are all surrounded by the gate oxide and the rectangular gate contact. It is conjecturable that the rectangular gate has a strong field-effect control ability to both the horizontal body and the two vertical parts due to the structure features mentioned above. And, the inner spacer actually prolonged the distance of the shortest path between source and drain contacts in the silicon which could eliminate the short channel effect that can not be avoided for multi-gate devices with planar channel features. Compared to other 3-D channel devices [21–24], the proposed structure needs no gate formation in the recessed region, which largely reduces the complexity of the inner structure of the recessed region.

**Table 1:** Parameter selection for RGUC FET in TCAD simulation

Parameters	Values
Body width (*W*)	6 nm
Vertical body thickness (*t*_bv_)	6 nm
Horizontal body thickness (*t*_bh_)	6 nm
Spacer thickness between S/D region (*t*_sp_)	0.5 to 4 nm
Vertical length of the gate (*t*_gate_)	8 to 16 nm
Gate oxide layer thickness (*t*_ox_)	1 nm
Extension height of spacer between S/D region (*h*_ex_)	0 to 10 nm
Inner height of spacer in the recessed region (*h*_in_)	3 to 10 nm
Doping concentration (*N*_D_)	1 × 10^17^ cm^−3^ to 2 × 10^18^ cm^−3^
Drain-source voltage (*V*_DS_)	0 to 1.0 V
Gate-source voltage (*V*_GS_)	0.4 to 1.0 V

## Results and Discussions

The Bohm quantum potential (BQP) model calculates a position-dependent potential energy term using an auxiliary equation derived from the Bohm interpretation of quantum mechanics. This model is derived from pure physics and allows the model to approximate the quantum behavior of different classes of devices as well as a range of materials. The effects of quantum confinement on the device performance, including *I*-*V* characteristics, will then be calculated to a good approximation. Previous studies show that the gate leakage current is negligible for cases of oxide thickness larger than 0.5 nm [7, 26].

**Fig. 2:**
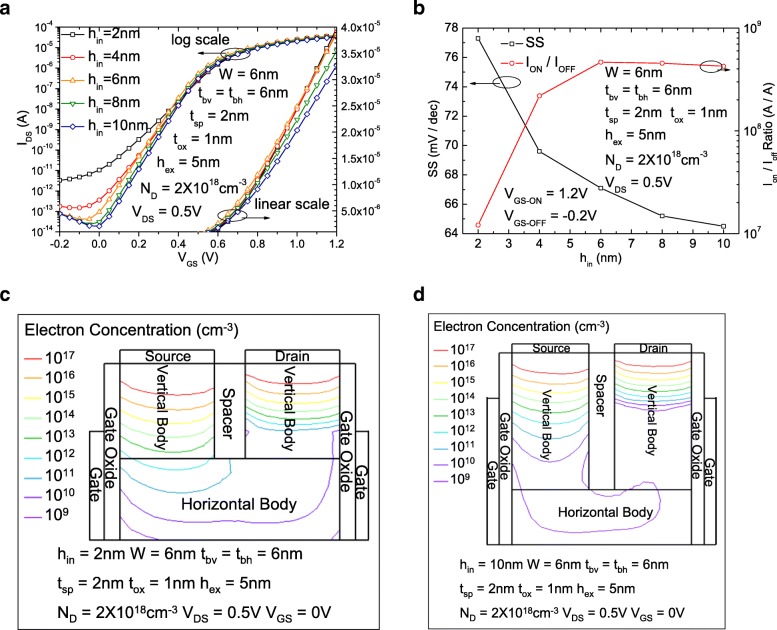
**a** The comparisons of the *I*_DS_-*V*_GS_ characteristics of the RGUC FET with different *h*_in_s on both logarithmic and linear scales. **b** The comparisons of subthreshold swings (SS) and *I*_ON_/*I*_OFF_ ratio of the RGUC FET with different *h*_in_s. **c** 2-D electron concentration distribution in the silicon body in off state for the device with 2-nm *h*_in_. **d** 2-D electron concentration distribution in the silicon body in off state for the device with 10 nm *h*_in_

Figure 2a shows the comparisons of the drain-source current gate-source voltage (*I*_DS_-*V*_GS_) characteristics of the RGUC FET with different *h*_in_s on both logarithmic and linear scales. Figure 2b shows the comparisons of subthreshold swings (SS) and *I*_ON_/*I*_OFF_ ratio of the RGUC FET with different *h*_in_s. With the increase of *h*_in_, the vertical path of the whole channel from source to drain is continuously increased, then the shortest effective channel length increases gradually, and the short-channel effect gradually weakens and is finally eliminated. The SS can realize a nearly ideal value of 65 mV/dec for *h*_in_ reaches 10 nm. The *I*_ON_/*I*_OFF_ ratio also increases about 35 times for *h*_in_ increases from 2 to 10 nm due to the continuously decreased SS. The prolonged *h*_in_ makes the distance of the shortest path from source to drain increases from 6 to 22 nm, which equals to 2 *h*_in_ + *t*_sp_ and is equivalent to the effective channel length of the proposed structure. Figure 2c and d show a 2-D electron concentration distribution in the silicon body in off state for the device with 2 nm and 10 nm *h*_in_, respectively. For the case of 2 nm, the highest electron concentration in the horizontal body region is about 10^12^ cm^−3^ and the distance between source/drain contact and the horizontal body region is very short. Thereafter, the source/drain bias seriously affect the electron distribution in the horizontal body region; the solution is to prolong the vertical channel which keeps the source/drain away from the horizontal body region. For the case of 10 nm, in Fig. 2d, we can see that the highest electron concentration in the horizontal body region is decreased down to 10^9^ cm^−3^, and it makes a more ideal fully depleted region for the off state which brings much lower level of leakage current.

**Fig. 3:**
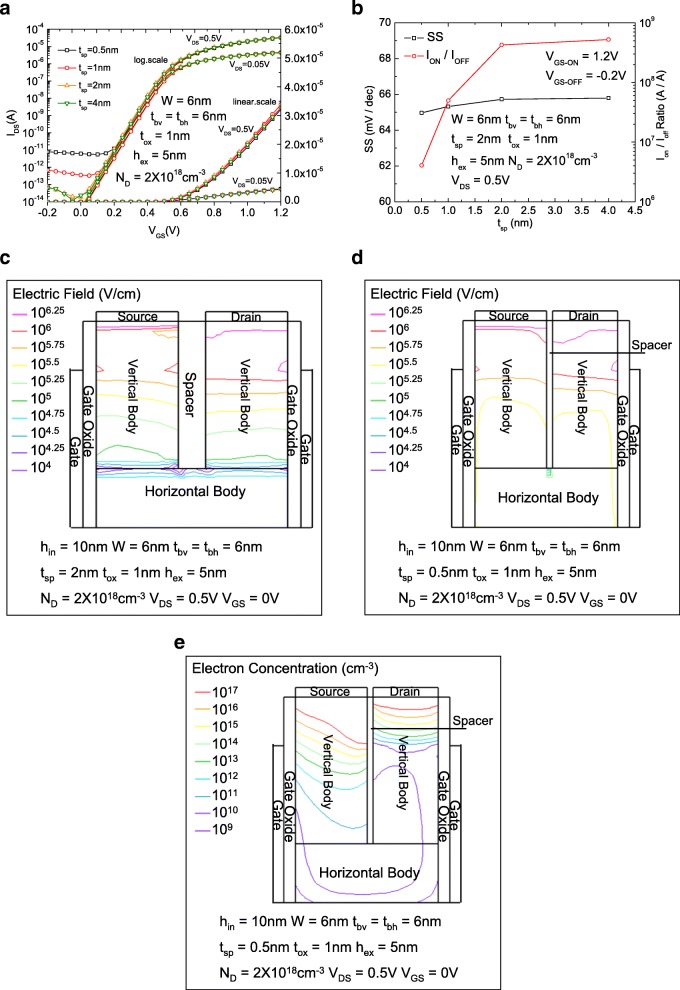
**a** The comparisons of the *I*_DS_-*V*_GS_ characteristics of the RGUC FET with different *t*_sp_s on both logarithmic and linear scales. **b** The comparisons of subthreshold swings (SS) and *I*_ON_/I_OFF_ ratio of the RGUC FET with different *t*_sp_s. **c** 2-D electric field distribution in the silicon body in off state for the device with 2 nm *t*_sp_. **d** 2-D electric field distribution in the silicon body in off state for the device with 0.5 nm *t*_sp_. **e** 2-D electron concentration distribution in the silicon body in off state for the device with 0.5 nm *t*_sp_

Figure 3a shows the comparisons of the *I*_DS_-*V*_GS_ characteristics of the RGUC FET with different *t*_sp_s on both logarithmic and linear scales. Figure 3b shows the comparisons of subthreshold swings (SS) and *I*_ON_/*I*_OFF_ ratio of the RGUC FET with different *t*_sp_s. With the decrease of *t*_sp_, the distance between source and drain contacts are continuously decreased too. The leakage current is mainly induced by band-to-band tunneling current. The tunneling probability is proportional to the band bending which can be equivalent to the electric field intensity in a certain point. The total tunneling current is the sum of the tunneling current generated in each point of the body region.

Figure 3c and Fig. 2d show a 2-D electric field distribution in the silicon body in off state for the device with 2 nm and 0.5 nm *t*_sp_, respectively. For a larger spacer thickness or a smaller drain-source voltage (*V*_DS_) bias, the electric field intensity on the interface between the spacer in the recessed region is not strong enough to produce a large amount of leakage current. The strongest electric field intensity appears near the interface between the gate oxide and the vertical body part, which is decided by *V*_GD_. However, if the source-to-drain distance is decreased to less than 1 nm (less than the gate oxide thickness), the strongest field intensity appears near the interface between the spacer in the recessed region and the two vertical body parts. It can be seen that when *t*_sp_ is less than 1 nm, for a larger *V*_DS_ (0.5 V for example), the leakage current is almost independent with the gate bias and mainly decided by the *V*_DS_. The SS is almost independent with *t*_sp_ and maintains a nearly ideal value of 65 mV/dec for a *h*_in_ = 10 nm case until *t*_sp_ is less than 2 nm. The *I*_ON_/*I*_OFF_ ratio maintains 10^8^ till *t*_sp_ = 2 nm and is seriously degraded for *t*_sp_ less than 2 nm due to the leakage current increase induced by the strong electric field appears near the interface between the spacer in the recessed region and the two vertical body parts. The electric field intensity of the silicon body in the body region is comprehensively enhanced for the 0.5 nm *t*_sp_ case. Figure 3e shows 2-D electron concentration distribution in the silicon body in off state for the device with 0.5 nm *t*_sp_. Compared with Fig. 2d, it is clearly seen that the electron concentration in the horizontal body region is enlarged from 10^9^ to 10^10^ cm^−3^. Besides, the dimension of 0.5 nm spacer thickness is very close to a single-molecule layer, which may cause damage of the insulation property of the spacer layer to some degree. Due to the reason mentioned above, the *t*_sp_ is suggested to be 2 nm for high-integration and low-leakage low-power consumption design.

**Fig. 4:**
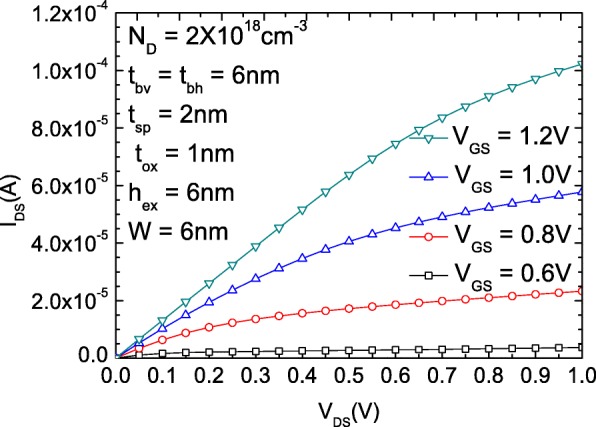
*I*_DS_-*V*_DS_ characteristic of the proposed RGUC FET with optimized device parameters

Figure 4 shows the *I*_DS_-*V*_DS_ of the proposed RGUC FET with optimized structure under different.

*V*_GS_ values, the SS of which is about 63 mV/dec, and the *I*ON/*I*OFF is 10^9^ ~ 10^10^. The saturated current increases as *V*_GS_ increases.

## Conclusions

A novel RGUC FET with high integration and high performance is proposed in this paper, which presents low-subthreshold swings and higher *I*_ON_/*I*_OFF_ ratio. The distance between source/drain (S/D) contacts can be reduced to 2 nm, with almost ideal characteristics such as SS, reverse leakage current, and *I*_ON_/*I*_OFF_ ratio. All the electrical properties are simulated with quantum models to ensure more precise results.

## Abbreviations

BQP: Bohm quantum potential
FET: Field-effect transistor
*h*_ex_: Extension height of spacer between S/D region
*h*_in_: Inner height of spacer in recessed region
*I*_OFF_: Off current
*I*_ON_: On current
JL: Junctionless
MOS: Metal oxide semiconductor
*N*_D_: Doping concentration
RGUC: Rectangular gate U channel
S/D: Source/drain
SS: Subthreshold swing
*t*_bh_: Horizontal body thickness
*t*_bv_: Vertical body thickness
*t*_gate_: Vertical length of the gate
*t*_ox_: Gate oxide layer thickness
*t*_sp_: Spacer thickness between S/D region
*V*_DS_: Drain-source voltage
*V*_GS_: Gate-source voltage
W: Body width
